# The Incidence Risk, Clustering, and Clinical Presentation of La Crosse Virus Infections in the Eastern United States, 2003–2007

**DOI:** 10.1371/journal.pone.0006145

**Published:** 2009-07-03

**Authors:** Andrew D. Haddow, Agricola Odoi

**Affiliations:** 1 Department of Entomology and Plant Pathology, The University of Tennessee, Knoxville, Tennessee, United States of America; 2 Department of Comparative Medicine, The University of Tennessee, Knoxville, Tennessee, United States of America; BMSI-A*STAR, Singapore

## Abstract

**Background:**

Although La Crosse virus (LACV) is one of the most common causes of pediatric arboviral infections in the United States, little has been done to assess its geographic distribution, identify areas of higher risk of disease, and to provide a national picture of its clinical presentation. Therefore, the objective of this study was to investigate the geographic distribution of LACV infections reported in the United States, to identify hot-spots of infection, and to present its clinical picture.

**Methods and Findings:**

Descriptive and cluster analyses were performed on probable and confirmed cases of LACV infections reported to the Centers for Disease Control and Prevention from 2003–2007. A total of 282 patients had reported confirmed LACV infections during the study period. Of these cases the majority (81 percent) presented during the summer, occurred in children 15 years and younger (83.3 percent), and were found in male children (64.9 percent). Clinically, the infections presented as meningioencephalitis (56.3 percent), encephalitis (20.7 percent), meningitis (17.2 percent), or uncomplicated fever (5 percent). Deaths occurred in 1.9 percent of confirmed cases, and in 8.6 percent of patients suffering from encephalitis. The majority of these deaths were in patients 15 years and younger. The county-level incidence risk among counties (n = 136) reporting both probable and confirmed cases for children 15 years and younger (n = 355) ranged from 0.2 to 228.7 per 100,000 persons. The southern United States experienced a significantly higher (p<0.05) incidence risk during the months of June, July, August, and October then the northern United States. There was significant (p<0.05) clustering of high risk in several geographic regions with three deaths attributed to complications from LAC encephalitis occurring in two of these hot-spots of infections.

**Conclusions:**

Both the incidence risk and case fatality rates were found to be higher than previously reported. We detected clustering in four geographic regions, a shift from the prior geographic distributions, and developed maps identifying high-risk areas. These findings are useful for raising awareness among health care providers regarding areas at a high risk of infections and for guiding targeted multifaceted interventions by public health officials.

## Introduction

La Crosse virus (LACV) is a member of the genus *Orthobunyavirus*, family Bunyaviridae, and is the causative agent of LACV infections. LACV was first isolated in 1964 [1], and has become one of most common causes of pediatric arboviral infections in the United States [2], [3]. The majority of LACV infections are transmitted to humans through the bite of the primary vector, the eastern tree-hole mosquito, *Aedes triseriatus* [4], [5]. The virus is maintained in nature through a vertical and horizontal transmission cycle involving *Ae. triseriatus* [6], [7], [8] and the primary amplification hosts: the eastern chipmunk, *Tamias striatus*, the gray squirrel, *Sciurus carolinensis*, and the fox squirrel *Sciurus niger* [9], [10].

LACV has traditionally been associated with forested areas in the upper-midwestern United States [11], but more recently as an emerging disease in the Appalachian region of the United States [12], [13], [14]. Unlike West Nile virus (WNV) infections that are more severe in the adults [15], the majority of severe LACV cases occur in children 15 years and younger [11], [16], [17], [18], with an average of 79 nationally reported cases per year since 1964, though pockets of higher infection risk have been reported [19]. The incidence risk in children 15 years and younger was believed to be 20–30 cases per 100,000, with a case fatality rate of less than 1 percent [13], [16], [17]. However, the true incidence of LACV infections are unknown as cases are typically under diagnosed, under-reported, and some are asymptomatic [18], [19], [20], [21]. In this study, we examined probable and confirmed cases of LACV infections from 2003–2007, to determine incidence risk, case fatality rates, and to assess the current spatial patterns of disease risk so as to identify those areas of highest risk for the implementation of future disease control strategies.

## Methods

### Study area

Our study area encompassed the eastern United States, the geographic region that includes the majority of previously reported LACV infections and the range of the primary vector, *Ae. triseriatus* [2], [16], [18], [22]. County level incidence risks were calculated and spatial analyses performed on 24 states in the study area. Seventeen of the 24 states reported probable and confirmed cases of LACV infections in children 15 years and younger (Table 1). The following states were part of the study area but did not report any LACV infections in children 15 years and younger during the study period: Arkansas, Delaware, Florida, Maryland, Missouri, New Jersey, and Pennsylvania. All of these states have reported LACV infections in the past.

**Table 1:** Non-Imported Probable and Confirmed Cases and Incidence Risk*of La Crosse Virus Infections in Children 15 Years and Younger Reported in the eastern United States, 2003–2007, by State.

State	Reported Cases	Percentage of Reported Cases	Incidence Risk by State	Range of Incidence Risk Among Counties Reporting Cases
West Virginia	83	23.4	57.8	7.5–228.7
Ohio	73	20.6	5.0	0.3–37.1
North Carolina	66	18.6	21.0	0.5–206.7
Tennessee	50	14.1	18.4	3.6–166.6
Wisconsin	21	5.9	12.1	1.1–76.7
Illinois	18	5.1	1.3	0.2–30.8
Minnesota	9	2.5	10.6	7.3–29.8
Virginia	8	2.3	7.8	1.6–35.2
Georgia	7	2.0	2.6	0.5–129.8
Indiana	6	1.7	2.0	0.5–20.6
Louisiana	4	1.1	6.5	4.0–23.2
Iowa	3	0.8	41.4	27.7–55.1
Kentucky	3	0.8	13.5	7.7–23.6
Michigan	1	0.3	1.0	1.0
Mississippi	1	0.3	8.3	8.3
Alabama	1	0.3	0.7	0.7
South Carolina	1	0.3	1.7	1.7
Florida†	–	–	–	–
Overall	355	100	7.2	0.3–228.7

* Incidence risk was calculated in counties reporting probable and confirmed cases of La Crosse virus infections and presented as the number of cases per 100,000 persons in children 15 years and younger, and are expressed here as a range in those states with two or more counties reporting cases.

† No incidence risk is reported for states not reporting cases 15 years and younger.

### Disease data

This study was conducted for all states reporting probable and confirmed cases of LACV infections in the United States, 2003–2007, through the ArboNET surveillance system [23]. The ArboNET surveillance system was established by the Centers for Disease Control and Prevention in 2000 to monitor the spread of West Nile virus in the United States. In 2003, the system was expanded to collect data on other arboviral diseases. Through ArboNET, participating health departments report human cases of arboviral disease. The ArboNET system was queried by the Centers for Disease Control and Prevention to search for all LACV infections.

Personal identifiers of patients were deleted before database construction. Clinical and epidemiological LACV data for 151 probable and 275 confirmed cases of LACV infections reported during this time period were provided by the Centers for Disease Control and Prevention. Cases that acquired infection outside of the county/state were excluded from spatial analyses (n = 10). This research was deemed exempt from review and certification by the University of Tennessee's Institutional Review Board following review by the Department of Entomology and Plant Pathology's Departmental Review Committee under the University of Tennessee's guidelines for research involving human subjects.

### Case definition

Confirmed cases of LACV infections are required to meet both the clinical and laboratory requirements set by the Centers for Disease Controls and Prevention's case definition for neuroinvasive domestic arboviral diseases [24]. This definition is reprinted below:

In the absence of a more likely clinical explanation as documented by a physician, confirmed cases must meet all of the following criteria:

**Clinical Criteria.** 1) Fever, AND

2) Acutely altered mental status, or other acute signs of central or peripheral neurologic dysfunction, or pleocytosis associated with illness clinically compatible with meningitis, AND

**Laboratory Criteria.** 3) a four-fold or greater change in virus-specific serum antibody titer, or isolation of virus from or demonstration of specific viral antigen or genomic sequences in tissue, blood, CSF, or other body fluid, or virus-specific immunoglobulin M (IgM) antibodies demonstrated in CSF by antibody-capture enzyme immunoassay (EIA), or virus-specific IgM antibodies demonstrated in serum by antibody-capture EIA and confirmed by demonstration of virus-specific serum immunoglobulin G (IgG) antibodies.

**Probable Case Criteria.** Cases that met the clinical definition and had stable (less than or equal to a two-fold change) but elevated titer of virus-specific serum antibodies, or virus-specific serum IgM antibodies detected by antibody-capture EIA but with no available results of a confirmatory test for virus-specific serum IgG antibodies in the same or a later specimen, are deemed probable.

### Population and geographic data

The 2005, United States Census Bureau, Estimated County Population Dataset [25], was used to calculate the total population and the population 15 years and younger for each county. These populations were used to provide the denominators for calculating incidence risk at the county level. As the United States Census Bureau does not provide yearly population estimates that include sex, age, and race for the county level, the 2000 United States Census [26] was used to provide the denominators for calculating the sex - age - and race - specific incidence risk for all counties reporting LACV infections. Geographic boundary files were downloaded from the United States Census, TIGER, Geodatabase [27], and used for all cartographic displays.

### Statistical and geographic analyses

Incidence risk was calculated and spatial analyses were performed on 123 probable and 232 confirmed cases 15 years and younger and on 151 probable and 275 confirmed cases for all ages occurring during the study period for which county level data were available (Table 1). Incidence risk was calculated for all counties in the study area (n = 1924) and for counties reporting both confirmed and probable cases of LACV infections under the age of 15 (n = 136) and for all ages (n = 161). Incidence risks were expressed as the number of cases per 100,000 persons.

To determine if there was a significant difference in incidence risk by month between states reporting cases in the northern (n = 7) and southern (n = 10) regions of the study area, we calculated the incidence risk by region and month using the both probable and confirmed cases 15 years and younger. The northern region was comprised of Illinois, Iowa, Indiana, Michigan, Minnesota, Ohio, and Wisconsin. The southern region was comprised of Alabama, Georgia, Kentucky, Louisiana, Mississippi, North Carolina, South Carolina, Tennessee, Virginia, and West Virginia.

All incidence risk computations and descriptive analyses were performed using STATA 10.0 [28]. Spatial empirical Bayesian (SEB) smoothing was used to adjust incidence risk due to spatial autocorrelation and high variances resulting from a small number of cases reported in some counties [29], [30], [31], [32]. The resulting smoothed incidence risks allow for better visualization of the spatial patterns compared to the unsmoothed risk.

Global Moran's I [33] and the Moran Local Indicators of Spatial Association (LISA) were used to assess for evidence of spatial clustering [34]. Statistical significance of both global and local Moran's I statistics were tested using 9999 permutations. All spatial analyses were performed using GeoDa Version 0.95i [35], and cartographic displays were done using ArcView GIS 9.2 [36].

## Results

### Descriptive analyses of cases

A total of 282 patients had confirmed LACV infections reported to the Centers for Disease Control and Prevention (Table 2). Most cases presented during July (24.9 percent), August (32.6 percent), and September (23 percent). Cases ranged in age from 0.5 to 86 years, with a median age of 9 years; 83.3 percent were under the age of 15 years, and the majority were males (64.9 percent).

**Table 2:** Epidemiological and Clinical Characteristics of Confirmed and Probable La Crosse Virus Infections Reported in the eastern United States, 2003–2007.

Variable	Total Confirmed (%)	Probable and Confirmed Cases Combined (%)
Sex		
Male	183 (64.9)	264 (60.6)
Female	99 (35.1)	172 (39.4)
Unknown	–	1
Age		
0.1–0.9 yr	3 (1.06)	5 (1.15)
1 yr	11 (3.9)	13 (2.98)
2–5 yr	59 (20.9)	98 (22.8)
6–10 yr	98 (34.8)	157 (26.4)
11–15 yr	64 (22.7)	87 (19.95)
16–20 yr	7 (2.5)	15 (3.44)
≥21 yr	40 (14.2)	61 (13.99)
Unknown	–	1
Race		
White	241 (95.3)	368 (95.6)
Black or African American	9 (3.6)	11 (2.86)
American Indian or Alaska Native	3 (1.2)	4 (1.04)
Asian	0 (0)	1 (0.259)
Other	0 (0)	1 (0.259)
Unknown	29	52
Month of presentation		
March	1 (0.35)	2 (0.458)
April	3 (1.06)	3 (0.686)
May	1 (0.35)	2 (0.454)
June	23 (8.2)	29 (6.64)
July	71 (24.9)	110 (25.2)
August	92 (32.6)	138 (31.6)
September	65 (23)	105 (24.0)
October	23 (8.2)	44 (10.1)
November	2 (0.71)	3 (0.686)
December	1 (0.35)	1 (0.229)
Clinical manifestation		
Meningioencephalitis	157 (56.3)	242 (55.3)
Encephalitis	58 (20.7)	78 (17.8)
Meningitis	48 (17.2)	87 (19.9)
Uncomplicated fever	14 (5.0)	18 (4.4)
Other	2 (0.7)	3 (0.7)
Unknown	3	8
Death	5 (1.86)	6 (1.43)
Unknown*	13	16

* Unknown, represents the number of confirmed and probable cases for which the case outcome was not reported.

The sex-specific incidence risk (per 100,000 persons) for all counties reporting confirmed cases was 1.9 and 1.0 for males and females, respectively. The age-specific incidence risk was 1.2 for children under one year; 5.4 for 1–5 years; 6.8 for 6–10 years; 4.7 for 11–15 years; 0.5 for 16–20 years; and 0.2 for 21 years and older. Blacks had the lowest race-specific incidence risk (0.3 per 100,000 persons) and American Indians had the highest (4.9 per 100,000 persons). The incidence risk for all counties reporting probable and confirmed cases combined in children 15 years and younger was 7.2 per 100,000 persons (mean 30.2), and 1.6 per 100,000 persons (mean 5.8) for the total population.

The incidence risk was significantly higher in the states in the southern region than those states in the northern region for June (p = 0.0018), July (p = 0.0002), August (p = 0.0033), and October (p = 0.0053) (Table 3). There was no statistically significant difference (p>0.05) in the incidence risk between the states in the northern and southern regions for the months of March, April, May, September, and November.

**Table 3:** Non-Imported Probable and Confirmed Cases and Incidence Risk* of La Crosse Virus Infections for Children 15 years and Younger in the eastern United States, 2003 to 2007, by Region.

	Northern Region··
Month	Incidence Risk
June	2.9
July	1.8
August	1.8
September	4.0
October	3.7
	Southern Region†
Month	Incidence Risk
March	2.4
April	16.7
May	20.7
June	10.7
July	8.9
August	9.8
September	12.6
October	8.5
November	2.0

* Incidence risk was calculated by region and month using the both probable and confirmed cases of La Crosse virus infections and presented as the number of cases per 100,000 persons in children 15 years and younger.

·· Illinois, Iowa, Indiana, Michigan, Minnesota, Ohio, and Wisconsin.

† Alabama, Georgia, Kentucky, Louisiana, Mississippi, North Carolina, South Carolina, Tennessee, Virginia, and West Virginia.

Clinically, the infections presented as meningioencephalitis (56.3 percent), encephalitis (20.7 percent), meningitis (17.2 percent), uncomplicated fever (5 percent), or other (0.7 percent) (Table 2). Deaths accounted for 1.9 percent of confirmed cases, and were reported in Indiana, Michigan, Tennessee, and West Virginia. All deaths occurred in patients suffering from LAC encephalitis (100 percent). Of those patients presenting encephalitis 8.6 percent died. The majority (80 percent) of these deaths were in patients 15 years and younger (median age 6, range 4–86) and occurred in males (80 percent). Three deaths occurred in the high risk clusters in West Virginia and in Tennessee.

### Spatial distribution

**Figure 1:**
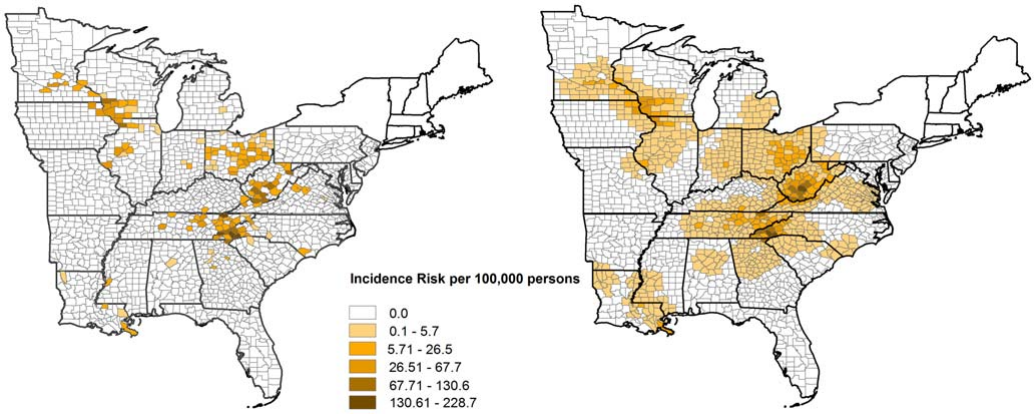
Distribution of unsmoothed and smoothed incidence risk in children 15 years and younger. The map on the left represents the distribution of a) unsmoothed risk, and on the right b) smoothed risk of La Crosse virus infections at the county level for the eastern United States.

**Figure 2:**
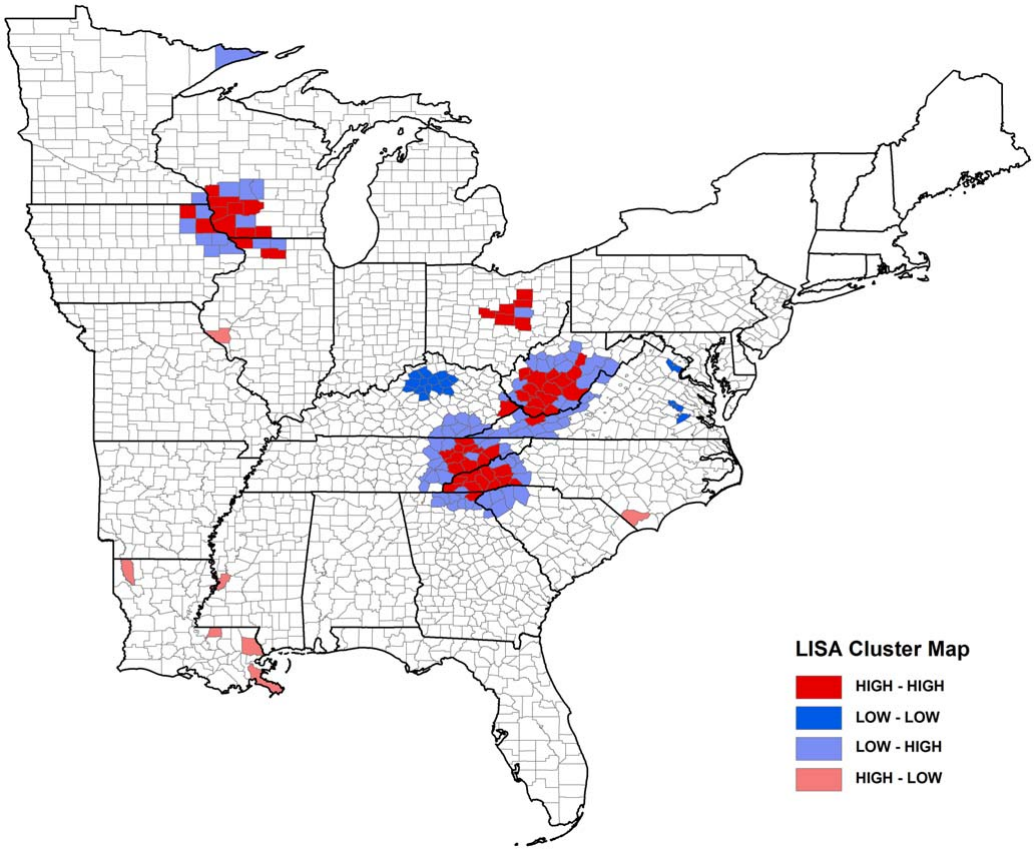
Spatial patterns of disease risk in children 15 years and younger. This map shows the significant clustering of La Crosse virus infections at the county level detected by the Moran's I Local Indicators of Spatial Autocorrelation (LISA) for the eastern United States. Four types of spatial autocorrelation are observed using the LISA statistic (High-High, Low-Low, High-Low, and Low-High). Positive spatial autocorrelation is represented by High-High and Low-Low, and negative spatial correlation by High-Low and Low-High. Positive spatial autocorrelation (i.e. an association of areas of similar values) were represented as either High-High (i.e. a high risk in an area surrounded by similarly high values in neighboring areas) or Low-Low (i.e. a low risk in an area surrounded by similarly low values in neighboring areas). Negative spatial autocorrelation (i.e. an association of areas of dissimilar values) was represented as either High-Low (i.e. a high rate in an area surrounded by low values in neighboring areas) or Low-High (i.e. a low rate in an area surrounded by high values in neighboring areas).

In children 15 years and younger (probable and confirmed combined), state-level incidence risk ranged from 0.7 to 56.4 per 100,000 persons whereas at the county-level incidence risk in counties reporting cases, ranged from 0.2 to 228.7 per 100,000 persons (Table 1). Geographically, the highest incidence risks were observed in western and central Illinois, northeastern Iowa, south central Minnesota, southwestern Wisconsin, and the Appalachian region (south central Kentucky, south central Ohio, western North Carolina, central and eastern Tennessee, south central West Virginia) (Figure 1). The spatial patterns are more easily recognizable when smoothed (Figure 1b) as compared to the unsmoothed incidence risk map (Figure 1a). The global Morans I value for children 15 years and younger, was 0.1904 (p = 0.0001) for only confirmed cases and 0.2223 (p = 0.0001) for both probable and confirmed cases combined. Forty-seven of the counties in the study area showed evidence of significant high incidence risk (p<0.05) detected by LISA using confirmed cases, while 54 counties had evidence of significant clustering of high risk (p<0.05) detected using both probable and confirmed cases. The spatial patterns for both confirmed cases and for probable and confirmed cases combined were similar. Therefore, only the spatial patterns using both the probable and confirmed cases are presented in Figure 2. The clusters detected by using both probable and confirmed cases combined were found in northeastern Iowa/southwestern Wisconsin, and in south central Ohio, western North Carolina/eastern Tennessee/northeastern Georgia, and south central West Virginia/eastern Kentucky/northwestern Virginia (Figure 2). The mean incidence risk for children 15 years and younger at the county-level for those counties that were part of the significant high clusters was 54.6 per 100,000 persons, and ranged from 4.7 to 228.7 per 100,000 persons.

## Discussion

Our study provides the first risk map of LACV infections for the United States, and presents insights into the clinical picture of LACV infections. We found both a higher incidence risk than previously reported ranging up to 228.7 cases per 100,000 persons in children 15 years and younger and a case fatality rate of 1.9 percent [5], [7], [8]. This study highlights the differences of the clinical presentation of LACV infections as meningioencephalitis, encephalitis, meningitis, uncomplicated fever, or other; rather than the traditional method of reporting of infections as purely LACV encephalitis. To our knowledge this is the first use of smoothing techniques, the global Moran's I, and the Moran Local Indicators of Spatial Association (LISA) to detect spatial clustering of LACV infections at a national level in the United States. We identified high risk clusters in four regions of the United States. These high risk clusters should be targeted for future studies and intervention efforts.

The majority of cases reported occurred in the summer months (June, July, and August) in agreement with previous studies [2], [17], [18], with a significantly higher incidence risk occurring in southern states during the months of June, July, August, and September. This is likely due to the transmission cycle of LACV, which involves both horizontal and vertical transmission. The burden of infected mosquitoes would continue to increase into the summer months as each successive generation of mosquitoes fed upon viremic amplification hosts, followed by increased transovarial transmission of the virus to their progeny. The highest risk of infection would thus occur during the height of this amplification process when the highest burden of infected mosquitoes would be reached. The height of this viral amplification coincides with the summer months when humans are most apt to spend the most time outdoors, thus putting themselves at an increased risk for coming into contact with infectious mosquitoes.

Temperature may play a role in the higher incidence risk in southern region of the United States. Given that transovarial transmission following one gonotrophic cycle is likely rare in nature [6], orally infected *Ae. triseriatus*, likely need to complete at least two gonotrophic cycles to transovarially transmit the LACV to their eggs [37]. As only a small percentage of *Ae. triseriatus* may survive to complete their second gonotrophic cycle in nature [37], an increase in the ambient temperature would increase the number of gonotrophic cycles possible in lifespan of a vector. Additionally, higher temperatures would have the added effect of increasing viral dissemination, titers, and transmission in vectors [38], [39]. Warmer temperatures, which are traditionally present for longer periods of time in the southern United States could therefore be playing a substantial role in increasing overall vectorial capacity.

Previous studies have shown that over 90 percent of reported cases occur in children 15 years and younger [16], with approximately 64 percent of these cases occurring in males [16]. Our study confirms these findings with roughly 83 percent of confirmed cases occurring in children 15 years and younger and 64.9 percent of these cases occurring in males. Clinical studies have demonstrated similar findings [13], [40], [41]. The tendency for children to develop severe LACV infections may be due to a variety of factors including differences in the pediatric and adult immune system, virus dose, and/or the longer time children may spend outdoors, though such risk factors are not well understood. It has been hypothesized elsewhere that the higher incidence risk in males may be due to the greater time boys spend outdoors, thus increasing their risk of contact with infectious vectors [21], [42].

Of the 426 reported probable and confirmed LACV infections during our study period, the Appalachian region including West Virginia, North Carolina, Ohio, and Tennessee reported 22.2 percent, 20.8 percent, 18.5 percent and 22.2 percent of reported cases, respectively, and accounted for 74.5 percent of all cases during the study period. A previous study from 1964–1981, reported 1348 cases in the United States of which 88.8 percent came from Ohio, Wisconsin, Minnesota, Illinois, and Iowa [16], while only 2 percent came from North Carolina, Tennessee, and West Virginia. Though our results encompass only a five-year period, the higher incidence risk in these states may indicate a shift in LACV infections from the upper Midwest to the Appalachian region of the United States [12], [13], [14]. The reason for this shift is unclear, but could be due to a number of factors including changes in diagnosis, reporting, prevention strategies, as well as changes in the epidemiology of the disease.

Though our finding of an mean incidence risk of 30.2 per 100,000 persons for children 15 years and younger in probable and confirmed cases combined for eastern United States was similar to previous reports of an incidence risk of 20 to 30 per 100,000 persons in the same age group [43], [44], our mean incidence risk in the significantly high spatial clusters was numerically higher, 54.6 per 100,000 persons, then the incidence risk previously reported for the same age group [43], [44]. In the individual counties reporting cases of LACV infections we found the incidence risk in children 15 years and younger ranging from 0.16 to 165.4 per 100,000 persons using only confirmed cases, and from 0.2 to 228.7 per 100,000 persons using both confirmed and probable cases combined. These results indicate the wide variation that occurs due to the focal nature of the virus within counties reporting LACV infections, and highlights the need to report the range of incidence risk, rather than reporting only the mean incidence risk which may not provide an accurate assessment of risk within these focal areas.

A recent study of the incidence of WNV infections in the United States by Lindsey et al. [45], analyzed five years of WNV infection data reported to the ArboNET surveillance system. The authors found a state level incidence risk for WNV infections ranging from 0.2 to 32.2 per 100,000 for the total population and a county level incidence risk ranging from 0.1 to 241.2 per 100,000 for the total population. Though the range of our state level incidence risk for LACV infections in the at-risk population was higher, ranging from 0.7 to 56.4 per 100,000 in children 15 years and younger, our county level incidence risk for LACV infections was similar to their findings, 0.7 to 228.7 per 100,000 persons in children 15 years and younger. These results indicate that the incidence risk of LACV infections are similar to that of WNV infections.

When the results from our analysis of those counties in the study area reporting both probable and confirmed cases of LACV infections are compared with pediatric WNV infection cases reported in Cuyahoga County, Ohio, in 2002 [15], LACV infections had a higher incidence risk in the pediatric population than that estimated for WNV infections, 7.2 per 100,000 persons in children 15 years and younger to 1.4 per 100,000 persons in children 5 to 17 years of age, respectively. In those counties reporting probable and confirmed cases of LACV infections in the population >16 years and older we found an incidence of 0.34 per 100,000 persons, while in Cuyahoga County, Ohio, LeBeaud et al. [15] estimated the incidence risk in the population ≥18 years and older to be 14.3 per 100,000 persons. It is clear that the severe infections caused by these two arboviruses present in the pediatric and adult populations differently, with severe LACV infections having a much higher incidence risk in children than adults, the opposite of that of WNV infections. The cause(s) for the differences in incidence risks between these two arboviruses within the pediatric and adult populations remains unknown, but warrants further investigation.

From the results of LACV serosurveys conducted in endemic regions it is clear that the risk of asymptomatic infections is much higher than symptomatic infections, with estimates of asymptomatic infections to clinical infections in pediatric populations ranging from 2∶1 to 1500∶1 [19], [20], [21]. The incidence risk of LACV infections are therefore likely much higher, as only the most severe cases typically present for medical care and hence get reported. The results from these studies suggest that there are most likely several hundred thousand infections per year in the United States [19], [20], [21], but our findings suggest that there are many more cases than previously estimated. The spatial distribution (Figure 1) reaffirms the focal nature of these infections, and identifies the areas with the highest risk.

The use of probable and confirmed cases combined increased overall cluster detection from 47 to 54 counties. The significant high risk clusters detected should be targeted for future studies and for interventions by public health officials. Reporting the mean incidence risk at the state or county level may lead to a distorted picture of the spatial patterns of LACV infections and thus decrease the perception of risk. Future reporting should include the range of incidence risk occurring in those counties reporting cases. This will minimize misperceptions of risk, as the use of incidence risk continues to remain the most used tool to identify high risk areas for education, prevention, and intervention.

Though LACV infections are typically reported as LAC encephalitis there appear to be four distinct clinical syndromes, as well as asymptomatic infections [2], [13], [40], [41], [46], These include the most severe LAC encephalitis, LAC meningioencephalitis, LAC meningitis, and LAC fever. It is difficult to assess the true incidence of these syndromes since they have often been collectively reported as LAC encephalitis in previous reports/studies [40], [41], [47]. Accurate reporting of the patients' clinical syndrome is necessary to determine and monitor the future rates of disease presentation.

Our study found a case fatality rate of 1.9 percent in confirmed cases, with all of the deaths occurring in patients presenting encephalitis. The majority of deaths were in children 15 years and younger. This finding is much higher than the case fatality rate of 0.3 percent reported in a previous study of LACV infections in the United States [16]. The reason for the higher case fatality rate is unclear, but many factors should be considered including variation in the virulence of the LACV strain(s) circulating in this region of the United States [48]. These findings suggest that LACV infections may be more severe than sometimes reported in medical literature. Three deaths (probable and confirmed cases combined) occurred in two of the four highly significant clusters of probable and confirmed cases of LACV infections in West Virginia and Tennessee.

Our study has some limitations. Probable and confirmed cases were reported through a passive surveillance system, which inherently suffers under-reporting. Not withstanding this limitation we feel that the majority of cases progressing to severe illness were diagnosed and reported to public health officials. Using only confirmed cases as well as probable and confirmed cases combined, we were able to demonstrate similar high incidence risk and case fatality rates. We were also able to show similar patterns in disease clustering. Clinical data was reported from multiple state health departments to the Centers for Disease Control and Prevention, which did not allow for the verification of laboratory results and the diagnosis of the specific clinical presentation for each patient. We feel that this is a small limitation and that the majority of cases reported have been correctly separated into the four manifestations of severe LACV infections by clinicians.

One drawback to using the LISA is the issue of multiple comparisons which increases type I error rates. We didn't make an attempt to adjust for this because some authors have suggested that any adjustments made to reduce the type I errors would increase type II errors [30], [49], in turn reducing the test's power to detect truly significant clusters.

Our findings of a high incidence risk within significantly high spatial clusters and high case-fatality rate indicate a much higher burden of disease than previously reported, and demonstrate that LACV infections are much more common than previously reported. We have demonstrated the usefulness of these spatial statistical techniques to detect hot-spots of infections, thus allowing for targeted interventions by public health officials while raising awareness among health care providers of geographic areas at the highest risk of disease.

Our results will allow focused national serological studies, form the basis for the development of predictive models of virus transmission, provide a methodology for the use spatial analyses at a national level for other infectious diseases, and demonstrate the need for the reporting of arboviral and other disease cases at smaller geographic scales.
